# New York Odontological Society

**Published:** 1888-02

**Authors:** 


### NEW YORK ODONTOLOGICAL SOCIETY.

Reported for the Independent Practitioner.

The January meeting of this society was held in the parlors of the New York Academy of Medicine, Tuesday evening, 10th ult., Dr. J. Morgan Howe, presiding.

Dr. R. R. Andrews, of Cambridge, Mass., read a paper on the "Development of Teeth, with Demonstrations of Dentine from the Odontoblasts and Fibril Cells." After elaborating his views concerning the formation of dentine, the doctor related his method of preparing specimens for microscopical slides. Those taken from the embryo are best for sections. He finds little or no satisfaction in the study or examination of specimens obtained by the ordinary methods of preparation, as he considers them imperfect. He thinks he has discovered two distinct varieties of cells that enter into the formation of dentine, one having a higher function than the other. These are the odontoblasts and the fibril cells. The former contribute the matrix and the latter complete the tissue. Dr. Andrews quoted from many noted histologists, presenting their theories of tooth and bone formation, and then gave the results of his own study and investigations. After reading his paper, he exhibited about fifty photo-micrographs which were beautifully projected upon a screen to illustrate the points under consideration.

Dr. Geo. S. Allen, referred, in a complimentary manner, to the photo-micrographs of Dr. Andrews, and reminded his hearers of the great, painstaking, persistent labor, and careful study required to produce such fine microscopical specimens. He, however, could not accept the theory that there are a double set of cells in tooth or bone formation. He thinks that the odontoblasts form both the matrix and fibrils.

*Dr. Andrews*—Thinks that he has fully demonstrated that there are two distinct sets of cells, each performing a different function, and he imagines that other histologists will, in time, arrive at the same conclusion.

*Prof. Carl Heitzmann*—Asserted that all dental fibrils arise from odontoblasts. He read from the INDEPENDENT PRACTITIONER extracts from a series of articles contributed by Drs. Heitzmann and Bödecker, and referred to illustrations accompanying said articles. These articles, he stated, were the result of eight years' careful study and investigation, and whoever had taken the trouble to read them ought to have acquired a fair idea of the formation of tooth structures. Prof. H. illustrated on the blackboard representations of cell and tooth formation, and declared that there were not two distinct sets of cells required to produce dentine or enamel.

*Dr. Allen*—Took exception to a statement of Prof. Heitzmann regarding enamel formation. He does not believe that a perfect cell, once formed, ever breaks up and transforms itself into any other tissue.

*Dr. Atkinson*—Quoted from John Hunter, that "inflammation is a return of tissues to their embryonic condition." We do not clearly understand the metamorphosis under which enamel is formed. Histologists are constantly laboring to correct each other's mistakes. We should learn all we can on this subject from the best of teachers, but be ready to admit that we do not yet know all.

On motion of Dr. Francis, a vote of thanks was tendered to Dr. Andrews by the society for his excellent paper and beautiful illustrations.

